# A Disgraceful West-End Club

**Published:** 1891-07-04

**Authors:** 


### The Doctor's Armchair.

### A DISGRACEFUL WEST-END CLUB.

When a man ceases to hate abuses it is with him a case either of softening of the brain or of deterioration of character. It is an abuse to attack venerable institutions which are honest and helpful to a country; it is also an abuse to perpetuate institutions which have ceased to be useful and have become injurious. "I send you," writes a correspondent, "a few lines on a case of hospital, or rather dispensary, abuse, which has come under my notice, as an example of what is actually going on at the present day in connection with some of the London medical institutions."

The story which our correspondent tells is a little off the usual lines of charity abuse. It deals with a West-end club, and lets in a peculiar light upon some of those institutions. The club in question is declared to be one of the richest and most prosperous of all the West-end clubs. In proof of this it is stated that it employs close upon a hundred waiters and attendants. The institution does not appear to be altogether destitute of a corporate conscience, for it gives a subscription to one of the leading dispensaries in its neighbourhood, and a handsome subscription too, amounting to no less than ten guineas a-year.

Now every well-conditioned man loves to see virtue rewarded, and we are quite sure that our readers will be glad to know that this generous club does not go without its *quid pro quo*. It is well known that there is a certain class of philanthropists whose leading principle is the acquisition of five per cent. for money invested in charitable objects. But those judicious and practical persons are simply nowhere in point of philanthropic wisdom and a high rate of interest compared with our West-end club. Here is a description of the reward of merit which is given to the corporate conscience that so generously subscribes ten guineas a year to a medical charity. The club, as we have said, employs about a hundred waiters and attendants. Any, or all, of these attendants can go to the dispensary and receive free advice from the members of the honorary medical staff as often as they please throughout the year. Not only do the whole hundred receive advice for nothing, but medicine also to any extent is given them without charge. Supposing each club servant to have no more than a single bottle of medicine and a box of aperient pills per annum, the whole of the ten guineas subscribed would, at ordinary chemists' charges, be more than swallowed up in the providing of physic alone. But when we remember the long hours, and the general character of the average club attendant, we cannot but feel that each servant is much more likely to have at least four or five bottles of medicine a year, with other accessories; and thus the club will get out of the dispensary in medicine alone, forty or fifty guineas a year in return for its ten.

All this is very bad, but it is nothing to what is yet to come. There is a resident medical officer at the dispensary; and if any one of the hundred servants of the club happens to be, or to think himself, too ill to go to the dispensary, the resident medical officer puts on his hat, with his stethescope inside it, and straightway proceeds to pay him a professional visit at the club. How many such visits are paid in the course of a year, and how much of the resident medical officer's time is thus taken up it is impossible to say; but the following statement of facts speaks volumes: "During the past few months," says our correspondent, "there has been, first, an outbreak of diphtheria, and, second, an epidemic of measles among the waiters. These, added to the recent epidemic of influenza, have given rise to a great many cases of illness, many of them requiring careful attendance for several days together." All of these have been visited at the club by the resident medical officer throughout their illnesses; all of them have had their medicine sent, and not a penny has been charged for either attendance or medicine from first to last.

All this medicine and attendance for ten guineas a year one would have thought would have satisfied even the corporate conscience of a rich West-end club. Nothing of the kind. "It appears," continues our correspondent, "that during the epidemic the steward of the club, a man receiving an income that many a medical man would be very thankful to earn by his profession, was attended gratuitously from the dispensary; nay, more, it is even stated that the late secretary was medically attended on the same terms."

The first thing that will occur to any honourable Englishman on reading this narration of facts is that it is disgraceful to everybody concerned: to the managers of the dispensary, to the honorary medical staff, and to their resident medical officer; to the hundred servants of the club, to the mean steward, the meaner secretary, and the conscienceless committee of the institution. If our correspondent is strictly correct as to his facts, the value of the medical work done, and of the medicines provided for the servants and other officials of this rich West-end club cannot have been less than a hundred pounds during the past six months alone. By way of payment for this medical work and these costly medicines the club gives five guineas, or a twentieth part of their value. The other nineteen parts are contributed by public subscription, partly by the annual guineas of the hard-working doctor or parson; partly by the poor clerk or small tradesmen who drops his shilling or his threepenny bit into the hospital box at the railway station, or at the street corner on the day of the Saturday Fund collection. But this is by no means the worst aspect of the case. The poor medical practitioners in the immediate neighbourhood of the club have been deprived of fees to the extent of at least ninety-five pounds during the six months, and that by the blind or wilful selfishness of their medical brethren who form the honorary staff of the dispensary.

We have no words wherewith to express our indignation. The members of the Club Committee ought to be pilloried, and the injured practitioners and poor subscribers to the dispensary should be supplied with unlimited rotten eggs for the occasion. After the Club Committee, the managers of the dispensary, and the members of the honorary medical staff should take their turn, and their punishment should be double that of the members of the Club Committee. One asks in despair what is to be done with educated men who show neither conscience nor sense? There are few questions more urgent than this among all the practical problems of the present time.

